# Suicide risk and REDCap: facilitating clinical trial inclusion

**DOI:** 10.1186/s12888-026-08128-4

**Published:** 2026-05-20

**Authors:** Benjamin O. Brenner, Molly Sullan, Molly E. Penzenik, Kelly A. Stearns-Yoder, Ryan Holliday, Claire A. Hoffmire, Lisa A. Brenner

**Affiliations:** 1 University of Colorado, Anschutz Medical Campus, Aurora, CO USA; 2 VA Rocky Mountain Regional Medical Center, Aurora, CO USA; 3 VA Brain Health Coordinating Center, Rocky Mountain Regional Medical Center, Aurora, CO USA; 4 Department of Physical Medicine and Rehabilitation, University of Colorado Anschutz Medical Campus, Aurora, CO USA; 5 VA Rocky Mountain Mental Illness Research, Education and Clinical Center (MIRECC) for Suicide Prevention, Aurora, CO USA; 6 VA Pacific Islands Health Care System, Honolulu, HI USA; 7 Departments of Psychiatry, Physical Medicine and Rehabilitation, University of Colorado Anschutz Medical Campus, Aurora, CO USA; 8 Departments of Physical Medicine and Rehabilitation, Psychiatry, and Neurology, University of Colorado, Anschutz Medical Campus, Aurora, CO USA

**Keywords:** Suicide risk, Clinical trials, At-risk cohorts

## Abstract

**Background:**

Considering increased rates of suicide, efforts have been focused on facilitating mental health research specifically dedicated to decreasing deaths by intentional self-harm. At the same time, notable suicide risk among those without identified mental health conditions highlight the importance of including such individuals in trials for a wide range of health conditions. To encourage enrollment of those at intermediate/high risk for suicide in both suicide prevention and more general health-related clinical trial research, a feasible and transferable strategy is needed. Such a strategy is outlined within this manuscript.

**Methods:**

The authors used Research Electronic Data Capture (REDCap), as well as validated measures, to monitor suicide risk among individuals participating in two clinical trials (Facilitating Assessment of At-Risk Sailors using Technology [FAAST]; Implementing Computerized Cognitive Behavioral Therapies [cCBT] for Suicide Prevention and Depression among At-Risk Rural and Urban Veterans). Individuals whose responses passed a predetermined threshold triggered an automated clinical alert, which resulted in clinical outreach by members of the study team who were licensed mental health providers. Descriptive data (e.g., number of outreaches, outreach attempt at which contact was made, number of individuals never reached) from each of these studies is presented in support of the feasibility of this approach.

**Results:**

During the FAAST trial, there were 55 instances in which a participant’s response met the clinical threshold for outreach based on responses to survey data (across 279 participants). Nearly all (*n* = 54 [98.2%]) of these instances resulted in a clinical outreach. In the cCBT trial, there were 200 instances (across 69 participants) in which a participant triggered a clinical alert. Outreach was triggered in 125 (62.5%) of these cases.

**Conclusions:**

Methods and findings highlight a feasible strategy for monitoring suicide risk among individuals participating in clinical trials. Use of these methods would be expected to facilitate the inclusion of individuals at intermediate and high suicide risk in a wide range of studies, while also prioritizing safety. Moreover, researchers should be encouraged to identify and disseminate methods, such as those outlined, to facilitate suicide prevention among diverse at-risk cohorts interested in engaging as study participants.

**Supplementary Information:**

The online version contains supplementary material available at 10.1186/s12888-026-08128-4.

## Introduction

Rates of suicide continue to be high among a wide range of cohorts (e.g., Service Members, Veterans, those living in socioeconomically disadvantaged areas) [1, 2]. In response, recent and important efforts have focused on facilitating research specifically dedicated to decreasing deaths by suicide [1, 3]. In a 2026 article, Interian and colleagues noted recent advances related to both health care system-wide prevention strategies and randomized clinical trials. However, suicide risk is related to diverse factors (e.g., mental health conditions, physical health conditions, environmental factors) and ongoing research efforts are warranted [2–4]. It is requisite that such research enroll and engage those at elevated risk for suicide to identify new and innovative means of reducing such deaths [3, 5, 6]. To ensure effectiveness when practices are implemented in real world settings, clinical trials specifically focused on suicide-risk mitigation must include those at intermediate and high (acute and chronic) risk for suicide, individuals that have traditionally been excluded, often due to concerns related to safety monitoring. As highlighted by Fisher et al. [6]. inclusion of those at increased risk poses important safety (e.g., risk assessment) and ethical issues (e.g., randomization) that must be addressed. The implementation of a dynamic risk assessment strategy throughout study participation is also supported by findings which highlight the limited utility of any specific evidence-based risk factor in terms of predicting an individual’s level of acute or chronic risk [7]. Unfortunately, literature regarding specific tools and processes that can be used by research teams to facilitate ongoing risk assessment using validated measures remains sparse [6].

This issue is not limited to mental health research. Work by members of this team and others continues to emphasize the role that physical health conditions and associated sequalae (e.g., decreased functioning) have on suicide risk. It is thus important to ensure both study inclusion and evidence-based risk management strategies to facilitate participation in potentially life extending or saving clinical trials related to primary health conditions. For example, work by members of this team suggests that Veterans with Amyotrophic Lateral Sclerosis (ALS), a progressive, degenerative motor neuron disorder which impacts functioning and significantly decreases life expectancy, are at increased risk for suicide [4]. In particular, risk appears to be elevated following receipt of this difficult diagnosis, with individuals presenting with increased risk for suicidal ideation. Reporting suicidal thoughts could thus result in exclusion from participating in clinical trials aimed at mitigating ALS symptoms, enhancing functioning, or extending one’s life. Similar assertions could be made among individuals living with other life threatening and/or chronic conditions such as head and neck cancer [8] or Parkinsons disease [9]. This challenge is particularly concerning for research with Veterans, given that members of this cohort are at increased risk for suicidal thoughts and behaviors [10], as well as for many chronic physical health conditions.

Towards this end, members of this team have identified a feasible and transferable strategy, using Research Electronic Data Capture (REDCap) [11], an accessible, low cost, and Health Insurance Portability and Accountability Act (HIPAA) compliant platform that is available to many researchers, including those at the Department of Veterans Affairs (VA), as well as validated measures and tools (e.g., Patient Health Questionnaire-9 [PHQ-9] [12]; University of Washington Risk Assessment Protocol [UWRAP]) [13] to monitor suicide risk among individuals participating in clinical trials. Information regarding the trials, participants, procedures, and feasibility data is further detailed below [14]. That is, within this manuscript, descriptive data is presented in support of the feasibility of this strategy.

## Methods

Research efforts outlined within this manuscript were reviewed and approved by all necessary regulatory bodies (i.e., Department of Defense, VA, local Institutional Review Board). REDCap [11, 14], as well as validated measures were used to monitor suicide risk among individuals participating in two clinical trials (Facilitating Assessment of At-Risk Sailors using Technology [FAAST] [15]; Implementing Computerized Cognitive Behavioral Therapies [cCBT] for Suicide Prevention and Depression among At-Risk Rural and Urban Veterans). Materials highlighting flow within REDCap [14] are included in supplemental materials to facilitate use of this tool by study teams (see Supplemental Materials. Selected Images of Clinical Monitoring REDCap Form). Information regarding participants to facilitate intervention in case of imminent risk (e.g., participant’s contact information and location) was pre-loaded in REDCap to facilitate outreach calls. Participants provided their consent to participate.

### Trials

Similar safety procedures to mitigate suicide risk were used in two studies led by members of this research team; one among United States Naval Surface or Aviation Force military Service Members (FAAST), and the other among Veterans with clinically relevant depression and or suicide risk (cCBT for Suicide Prevention and Depression). Additional information regarding the clinical and safety procedures is provided below. FAAST, a randomized controlled trial was implemented to evaluate, Cogito Companion, a secure, privacy-compliant mobile app versus an active control, as an upstream suicide prevention [15]. The study participant population consisted of Sailors. The second trial, cCBT for Suicide Prevention and Depression is being implemented to test the acceptability and feasibility of two computerized interventions among Veterans eligible to seek care within the Veterans Health Administration with suicidal thoughts and behaviors or symptoms of depression. For more specific information regarding each of the clinical trials see ClinicalTrials.gov (FAAST: NCT04159480; cCBT for Suicide Prevention and Depression: NCT06375083). The FAAST trial has ended and the cCBT trial is closed to recruitment.

### Tool

REDCap [11, 14] is a secure compliant (21 Code of Federal Regulations Part 11, Federal Information Security Modernization Act, HIPAA, General Data Protection Regulation) web-based application to manage online surveys and databases [14]. 

### Measures

Beck Scale for Suicide Ideation (BSS) [16]. The BSS is a self-report assessment measure to identify individuals at risk for suicide. Items 1–5 were used to monitor risk.

The Columbia-Suicide Severity Rating Scale (C-SSRS) Screener [17] is a screening measure, administered by a clinician or electronically, that assesses for suicidal ideation and behaviors based on established definitions. The C-SSRS screener queries regarding intensity of suicidal ideation, specifically asking about frequency, duration, intrusiveness, controllability, and deterrents, as well as suicide-related behavior, such as attempts.

Patient Health Questionnaire-9 (PHQ-9) [12]. The PHQ-9 is a 9-item, self-report rating inventory that measures symptoms related to depression. In specific, responses to item 9 were monitored: “Over the last 2 weeks, how often have you been bothered by any of the following problems? Thoughts that you would be better off dead or of hurting yourself in some way.”

University of Washington Risk Assessment Protocol-Revised (UWRAP) [13]. The UWRAP was used during clinical outreach phone calls to facilitate risk identification (cCBT trial). The UWRAP has been recommended by the National Institute of Mental Health and has been used in over 20 years of research. In specific, items 16, 17, and 20 from the Pre-assessment section were implemented.

### Automated risk assessment protocols and follow-up procedures

#### FAAST

Sailors completed weekly surveys, and responses were monitored during business days (excluding holidays and weekends). Survey items that prompted an outreach included: (1) PHQ-9 item 9 [12] - a response of “2” [more than half the days] or “3” [nearly every day]; (2) BSS [16] - any response of “2” on items 1–4 or 5 (criteria changed over the course of the study); and/or (3) any positive response on items 3–6 on the C-SSRS Screener [17]. In addition, for those in the Cogito group if scores on measures were below threshold for outreach, but dashboard data [collected via the app] was concerning, outreach was considered (data regarding outreach secondary to dashboard data is not reported in this manuscript). In all cases, if the participant met the clinical threshold, they received an outreach call from a licensed mental health clinician (i.e., licensed psychologist or clinical social worker), as well as automatic notification from MyCap, a participant-facing mobile application, with resources (e.g., Military and Veterans Crisis Line, Military OneSource, and Military Health System provider contact information [individualized data entered by participant or study team]). During the outreach call, information was gathered including coping strategies, reasons for living, and assessment of physical/mental health symptoms that may be eliciting suicidal ideation, intent, and/or a plan. If appropriate and/or desired, referral assistance for clinical care was provided. All information gathered during the clinical call, as well as app monitoring data (Cogito group only), was used to determine level of risk and appropriate actions to be taken. Three risk levels were used: (1) imminent acute risk; (2) increased acute risk; or (3) no perceived increased acute risk. Procedures for additional follow-up were made based upon level of risk.

#### cCBT

**Fig. 1:**
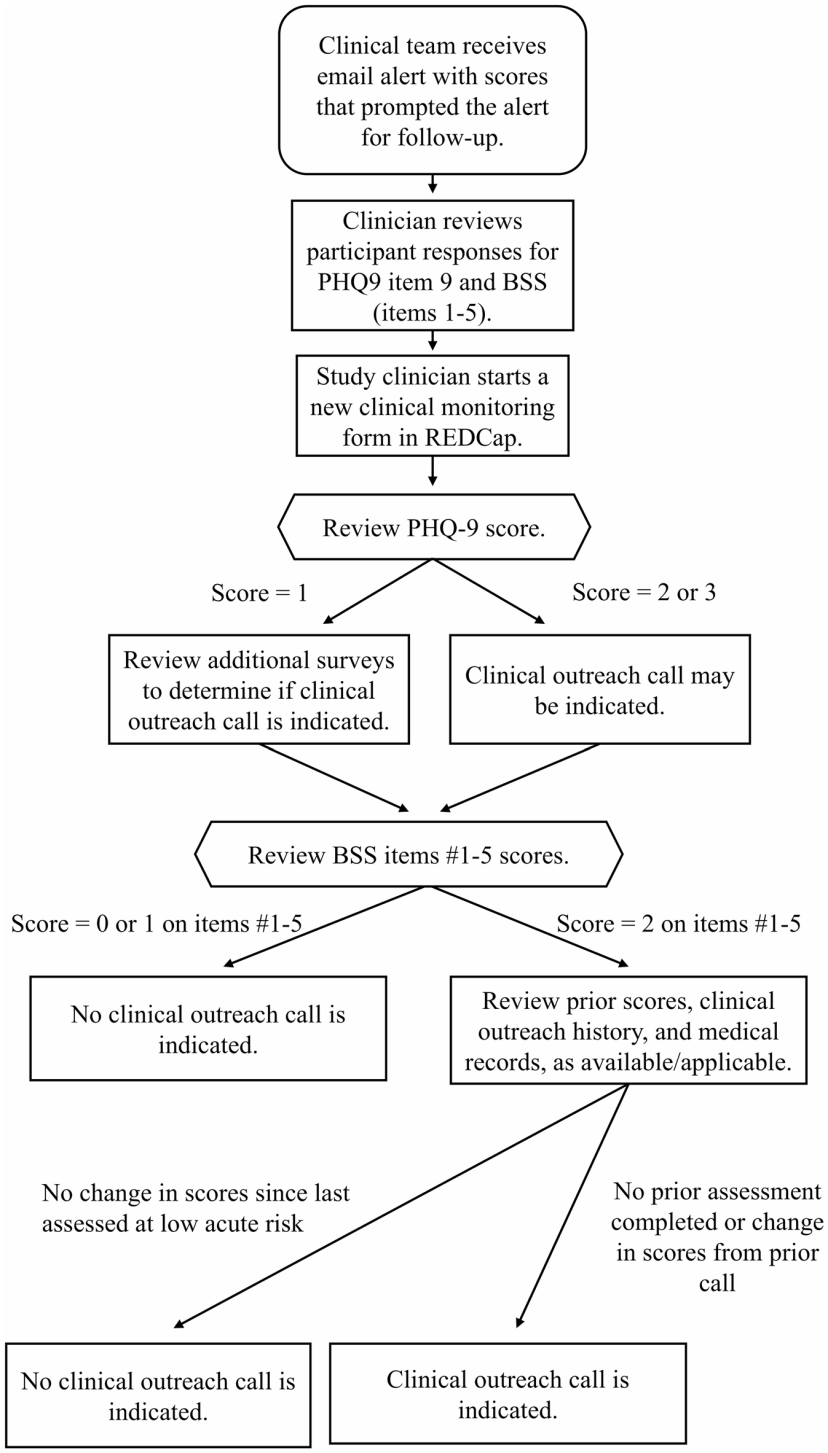
cCBT automated risk workflow. Rounded rectangles represent a clinical state or condition; Hexagons represent a decision point - formulated as a question that can be answered “Yes” or “No”. Rectangles represent an action in the process of monitoring

Weekly automated clinical monitoring was conducted based on responses to the PHQ-9 and BSS. Participant survey responses to the PHQ-9 (Item 9) and BSS (Items 1–5) were monitored during each business day by research team members who were licensed study clinicians. If participants’ responses met the clinical threshold (i.e. PHQ-9 response of “2” [more than half the days] or “3” [nearly every day] on PHQ-9 item 9, or BSS response of “2”) they received an outreach call. Lack of responses to surveys may have also prompted clinical/administrative outreach. See Fig. 1: cCBT Automated Risk Workflow.

**Fig. 2:**
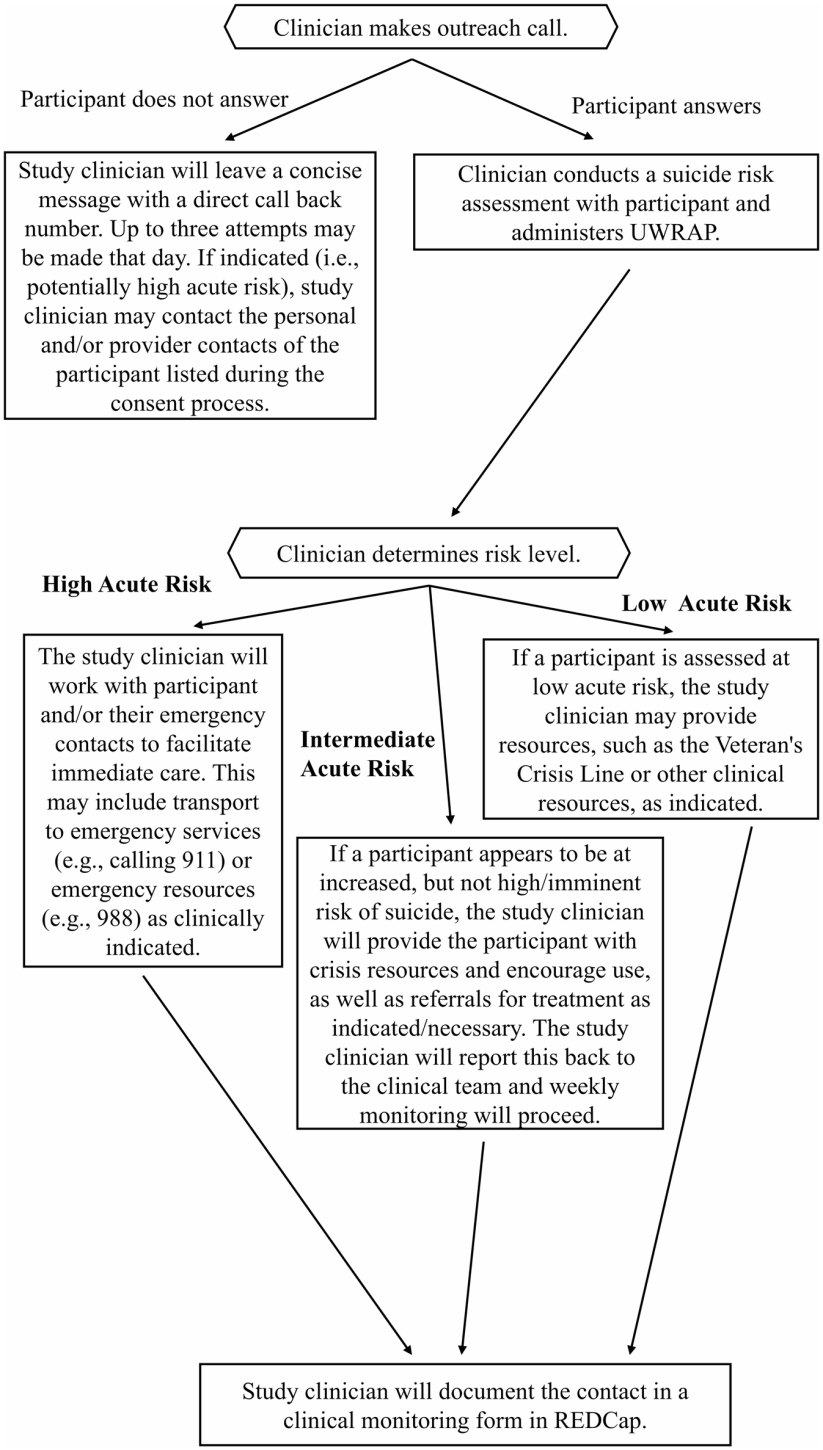
cCBT clinical outreach workflow. Rounded rectangles represent a clinical state or condition; Hexagons represent a decision point - formulated as a question that can be answered “Yes” or “No”. Rectangles represent an action in the process of monitoring

To facilitate outreach, a REDCap report of survey responses above the clinical threshold (clinical alert) was sent to study team members, with the study clinician then following the outreach workflow (see Fig. 2. cCBT Clinical Outreach Workflow) to facilitate risk assessment. The study clinician assigned to monitor that day would review survey responses via the email and/or REDCap to make a clinical determination as to whether an outreach was necessary. Specifically, on the first instance that a participant alerted, the study clinician would review responses and if indicated conduct a brief medical record review to obtain relevant history (e.g., involvement in ongoing care with a mental health provider, determine whether suicide risk assessments and interventions such as a safety plan had been completed, and other relevant evidence-based risk/protective factors [1] prior to calling). During the outreach call, the study clinician stated the reason for their call and conducted a clinical assessment for suicide risk. Additionally, the study clinician administered items 16, 17, and 20 on the pre-assessment UWRAP [13],which included questions regarding the individuals urge to self-harm, kill themselves, and/or harm another person. The study clinician documented responses, and made a risk determination (i.e., low, intermediate, or high acute risk). Resources were offered or provided as indicated (e.g., help outreaching their primary care or mental health provider). Further, if the participant reported any difficulty with the intervention itself, this would be documented and reported back to the study team. All clinical outreach calls were documented in REDCap in a clinical outreach monitoring form.

If during the first outreach call a participant was assessed at low acute risk, survey results were considered baseline and further outreach occurred if deviation from baseline was observed. Specifically, if there was an increase in scores, an additional clinical outreach call may have occurred based on clinical judgement, chart review, review of prior responses, and/or clinical notes from previous outreach calls. If during the initial call, a participant was assessed at moderate or high acute risk, and survey scores were above the above stated threshold, they continued to receive additional check-in calls from the study clinician, as clinically indicated/appropriate, until scores reached low acute risk. A new clinical monitoring form was completed each time a survey alert occurred to document the content of the outreach call and/or outline the reasoning for not conducting the outreach call that week.

### Data analyses

Variables were summarized as means or number and percent.

## Results

### FAAST

During the FAAST trial, there were 55 instances for which a participant’s response to one or more risk screening measures met the threshold for outreach, triggering a clinical alert. These 55 instances represented 41 unique participants (average of 1.3 clinical alerts per participant) Nearly all (*n* = 54 [98.2%]) of these instances resulted in a clinical outreach. For the single case where the participant was not outreached, the clinical team member determined that there was no perceived acute risk. One additional outreach was made to a participant even though their survey responses for that week did not meet the threshold, based on the participant’s pattern of responses to earlier surveys.

Up to three attempts were made to contact participants following a clinical alert. For the 55 instances in which a clinician determined an outreach was necessary, contact was made with 69.1% (*n* = 38). Eighteen (32.7%) outreaches resulted in contact with the participant on the first attempt. Of the 38 cases where contact was made, the study clinician determined the participant’s to be at “Increased Acute Risk” in 18 (47.4%) cases, and at “No Perceived Acute Risk” in 20 (52.6%) of the cases. No participants were determined to be at “Imminent Acute Risk.” For additional data regarding patient outreach (e.g., number of outreaches, outreach attempt at which contact was made, number of individuals never reached) see Table 1. Survey alerts by week are reported in Table 2.

**Table 1:** Alert and outreach summary data by study

	FAAST Study (*n* = 279 Participants)	cCBT Study (*n* = 69 Participants)
Number of survey alerts	55†	200
Number of attempted outreaches	55 (100%)	125 (62.5%)
Number of attempted outreaches where contact was made	38 (69.1%)	89 (71.2% of attempted outreaches)
Number of outreaches where contact was made on the first try	18 (32.7%)	73 (58.4% of attempted outreaches where contact was made)
Number of outreaches where contact was made on the second try	11 (20%)	12 (9.6% of attempted outreaches where contact was made)
Number of outreaches where contact was made on the third try	9 (16.4%)	4 (3.2% of attempted outreaches where contact was made)
Number of attempted outreaches where contact was never made	17 (30.9%)	36 (28.8% of attempted outreaches)
Clinician attempted contact 1 time, did not reach, did not attempt again*	1 (1.8%)	30 (83.3% of attempts where contact was never made)
Clinician attempted contact 2 times, did not reach, did not attempt again*	0 (0%)	4 (11.1% of attempts where contact was never made)
Clinician attempted contact 3 times but never reached**	16 (29.1%)	2 (5.6% of attempts where contact was never made)

† In addition to risk identification via survey response, clinicians also used a separate proprietary tool unique to the FAAST protocol to inform outreach. Outreach calls prompted by the propriety dashboard data are not included here. *At times additional data was received by the study team that suggested that no additional outreach was indicated. In other cases, the participant’s mailbox was full or not set up, and scores did not indicate imminent risk.**Per protocols 3 attempts were made to outreach patients unless imminent risk was noted

**Table 2:** Survey alerts by week

	FAAST Study (*n* = 279 Participants; 55 Total Survey Alerts)	cCBT Study (*n* = 69 Participants; 200 Total Survey Alerts)
Baseline	20 (36.4%)	**N/A**
Week 1	N/A	45 (22.5%)
Week 2	10 (18.2%)	26 (13.0%)
Week 3	N/A	26 (13.0%)
Week 4	7 (12.7%)	18 (9.0%)
Week 5	N/A	16 (8.0%)
Week 6	8 (14.5%)	7 (3.5%)
Week 7	N/A	9 (4.5%)
Week 8	4 (7.3%)	9 (4.5%)
Week 9	N/A	7 (3.5%)
Week 10	3 (5.5%)	6 (3.0%)
Week 11	N/A	7 (3.5%)
Week 12	2 (3.6%)	3 (1.5%)
Week 13	N/A	2 (1.0%)
Week 14	N/A	7 (3.5%)
Post-Intervention	1 (1.8%)	12 (6.0%)

### cCBT

In the cCBT trial (which is ongoing), there were 200 instances (across 69 participants) in which a participant triggered a clinical alert (average of 2.9 alerts per participant). The study clinician determined that an outreach was warranted in 125 (62.5%) of these cases. As highlighted above, if it was determined that risk was relatively stable, additional outreach calls were not conducted.

In the 125 instances requiring outreach, 89 (71.2%) resulted in contact during one of the three attempts. Seventy-three (58.4%) outreaches made contact with participant on the first attempt. Of the 89 cases where contact was made, the study clinician assessed one participant (1.1%) to be at “High Acute Risk”, five (5.6%) to be at “Intermediate Acute Risk”, and 83 (93.3%) to be at “Low Acute Risk”. For additional data regarding patient outreach see Table 1. Survey alerts by week are reported in Table 2.

## Discussion

Although both studies were suicide prevention focused, cohorts and treatment targets varied. As expected, the number of outreaches triggered differed between studies. In general, the rate of increased suicide risk among participants would be expected to vary between studies depending on a wide range of factors (e.g., focus of intervention, population of interest); thus safety monitoring implications would also be expected to vary. Nevertheless, findings highlight that the strategy outlined was feasible when implemented among these two different cohorts. Moreover, even among those in the cCBT trial, a group at higher risk by design, most individuals were identified as being low risk at clinical outreach. As such, most outreach calls were brief, with participants not requiring referral for additional urgent or crisis-related services. When contacting participants, efforts were also made to limit recommended interventions outside of FAAST or cCBT. That is, outreach procedures were constructed in a manner to preserve the trial design, while ensuring participant safety. As such, in most cases, participants were reminded of existing resources (e.g., strategies for contacting their primary mental health provider, Veteran Crisis Line) versus new resources.

As these procedures were implemented in support of larger trials, methods of implementation (e.g., feasibility outcomes) were certainly not as rigorous as those employed to evaluate the aims of the studies. Nonetheless, similar procedures were implemented among two cohorts being studied in very different settings. The goal of publishing these findings was not to provide the most reliable and accurate feasibility data regarding these procedures. In addition, the findings should not be interpreted to suggest that this is the only way to standardized monitoring for suicide risk. Alternately, considering the importance of including those at risk for suicide in a wide-range of trials, it is recommended that teams explore implementing similar procedures. Moreover, it is worth highlighting that several participants triggered numerous outreach calls. In cases where research teams are having frequent contacts with individuals, teams may want to consider the clinical impact of such calls (research participation effects), and evaluate outcomes accordingly [18]. Finally, additional work to describe outcomes during the study period (e.g., suicide attempts) and validate strategies such as the one outlined in the manuscript are encouraged.

An inherent limitation of this study is the reliance on measures which query traditional risk factors. Work by multiple teams has highlighted the dynamic nature of suicide risk, as well as the importance of leveraging multiple data points and advance methods (e.g., machine learning, ecological momentary assessment) to facilitate prediction [7, 19, 20]. For example, Rudd and Bryan highlight the utility of assessing constructs other than suicidal ideation (e.g., “Do you ever feel like your problems are unsolvable?”) [19]. Certainly, as such work progresses, information could be collected at baseline and during the study period to evaluate risk and facilitate outreach using various evidence-based means.

## Conclusion

Use of methods such as those outlined in this manuscript is expected to facilitate the inclusion of individuals at increased risk for suicide in clinical trials, while also prioritizing safety. Although the trials outlined above were related to mental health, it is expected that these methods could also be implemented in the context of trials focused on physical health conditions; thereby increasing the generalizability of findings.

## Supplementary Information

Below is the link to the electronic supplementary material.

Supplementary Material 1

## Data Availability

Based on VA policies the datasets generated and/or analyzed during the current study are not publicly available.

## Abbreviations

ALS: Amyotrophic Lateral Sclerosis
BSS: Beck Scale for Suicide Ideation
C-SSRS: Columbia-Suicide Severity Rating Scale
cCBT: Cognitive Behavioral Therapies
VA: Department of Veterans Affairs
FAAST: Facilitating Assessment of At-Risk Sailors using Technology
HIPAA: Health Insurance Portability and Accountability Act
PHQ-9: Patient Health Questionnaire-9
REDCap: Research Electronic Data Capture
UWRAP: University of Washington Risk Assessment Protocol
