# Supplementary material for: Suicide risk and REDCap: facilitating clinical trial inclusion
Source: BMC Psychiatry. 2026 May 20;26:592. doi: 10.1186/s12888-026-08128-4 (PMC13445734; doi:10.1186/s12888-026-08128-4)
Supplement: Supplementary file 1 [file 12888_2026_8128_MOESM1_ESM.docx]

**Supplemental Materials:** **Selected Images of Clinical Monitoring REDCap Form.**

1. **Setup (repeating instances enabled)**

2. **Reviewer and Survey Information.**

3. **Participant’s Contact Information and Location and Details about Outreach Attempt(s).**

4. **Risk Assessment and Associated Documentation.**
